# Supplementary material for: Natural hybridization in heliconiine butterflies: the species boundary as a continuum
Source: BMC Evol Biol. 2007 Feb 23;7:28. doi: 10.1186/1471-2148-7-28 (PMC1821009; doi:10.1186/1471-2148-7-28)
Supplement: Additional File 1 — Hybrids between species of Heliconius and Eueides butterflies: a database. HTML file linking to database of all known wild-caught interspecific hybrid specimens in the Heliconiina, consisting of introductory text, a list of specimens, together with collection data and photographs of the specimens, and links to information about some artificial hybrids and mutants in the group. This is an edited copy of our online database of Heliconius hybrids [102]. To view database, download zip file and extract to a separate folder, then open index.html within that folder. [file 1471-2148-7-28-S1.zip › hecele02.html]

hybrid hecele02


---


Hybrid between *Heliconius hecale versicolor* and *H.
elevatus pseudocupidineus*
Peru
Left: © Andrew V.Z. Brower; Right: © Gerardo Lamas

Return to table
of hybrids

To next hybrid
  
To previous hybrid

```
NOTES

No:                      15
Genus of species 1:      Heliconius
Species 1:               hecale
Subspecies of species 1: versicolor?
Genus of species 2:      Heliconius
Species 2:               elevatus
Subspecies of species 2: pseudocupidineus
Sex:                     m
Country:                 Peru
Locality:                Loreto: Yurimaguas
Year:                    1919
Photo no.:               hecele02
Named hybrid:            f. noeldneri Neustetter (holotype)
Collection:              Wien
Collector:               O. Michael
Author/publication:      Holzinger & Holzinger 1994: 27,1b
Notes:                   BC->elevatus? Suspected on basis of illustration & visit by Andrew Brower; hecale as parent suggested by regular spacing of apical spots and scalloped hindwing margin
```

**Last updated:** 18 October 2003

---
